# Epidemiology of Injuries in High-Performance Taekwondo: A Systematic Review

**DOI:** 10.7759/cureus.93356

**Published:** 2025-09-27

**Authors:** Celia García Blanco, Aldara Vazquez-Mendez, Orlando Conde, Irimia Mollinedo-Cardalda

**Affiliations:** 1 Faculty of Physiotherapy, University of Vigo, Pontevedra, ESP; 2 Faculty of Physiotherapy, Isaúde Research Group, University of Vigo, Pontevedra, ESP

**Keywords:** athletic injuries, epidemiology, martial arts, sports injuries, taekwondo

## Abstract

Taekwondo is a traditional South Korean martial art and Olympic combat sport that has gained global popularity. At elite competitive levels, its high-intensity, full-contact nature carries a substantial risk of injury. The aim of this study was to synthesize current evidence on the epidemiology of injuries in high-performance taekwondo athletes and to inform future injury-prevention strategies. Following Preferred Reporting Items for Systematic Reviews and Meta-Analyses (PRISMA) 2020 guidelines, a systematic search was conducted in PubMed, Medline, Scopus, Web of Science, CINAHL, and SportDiscus (February-March 2025). Search terms included Medical Subject Headings (MeSH) “Wounds and Injuries,” “Athletes,” and “Martial Arts,” as well as the free term “taekwondo,” combined with Boolean operators. Eligible studies were observational, published in English or Spanish between 2018 and 2024, and involved elite taekwondo athletes. Data extraction and methodological quality assessment were performed independently by two reviewers. Quality was appraised using the Joanna Briggs Institute (JBI) critical appraisal checklist for prevalence studies. After conducting the research, eight studies met the inclusion criteria. Lower limb injuries - particularly to the foot and knee - were the most common. Contusions predominated, followed by sprains and ligament tears. Direct contact was the leading cause of injury; overuse was more frequent in training settings. Most injuries were of low severity, resulting in one to seven days of time loss. Incidence rates varied due to heterogeneous denominators (athlete-exposures, minutes, or hours) but were generally higher during competition. Methodological quality ranged from moderate to high, with frequent shortcomings in bias control and standardized outcome measurement. In conclusion, high-performance taekwondo athletes are most prone to lower limb injuries, especially contusions from direct contact in competition. Preventive programs should focus on lower limb protection, impact absorption, and overuse reduction. Future research should standardize injury definitions, denominators, and reporting methods to enhance comparability and inform evidence-based prevention strategies.

## Introduction and background

Taekwondo is a traditional South Korean martial art and full-contact Olympic combat sport characterized by dynamic kicking and striking techniques. Since its establishment as a global sport under World Taekwondo (WT) in 1973, its popularity has grown to an estimated 80 million practitioners across more than 200 countries [1]. It debuted as a demonstration sport at the 1988 Seoul Olympics and became an official medal event at the Sydney 2000 Olympics [1].

Elite-level taekwondo demands high levels of agility, speed, flexibility, coordination, and endurance, with frequent explosive movements and directional changes. Together with its full-contact nature, these demands increase the risk of acute and overuse injuries, particularly in the lower limbs [2]. While participation offers significant physical and psychological benefits - including improved muscle strength, balance, cardiovascular fitness, and stress management - it also brings substantial injury risks inherent to high-performance sport.

Taekwondo is contested in three disciplines: demonstration, poomsae (forms), and sparring. The Olympic sparring format involves two athletes in the same weight category scoring via torso or head strikes. The sport's injury profile has evolved since the introduction of the Protector and Scoring System (PSS) with electronic body protectors in 2012 and electronic headgear in 2017, which altered match dynamics, reduced head impact forces, and may have influenced injury patterns [1].

Drawing from broader sports literature, injury incidence in elite and high-performance athletes typically ranges between 1.9 to 15.5 injuries per 1,000 athlete-exposures in field and team-based sports such as soccer or rugby [3]. However, in combat sports, higher rates have been recorded - 79.4 injuries per 1,000 athlete-exposures in taekwondo and up to 229 in mixed martial arts (MMA) [4].

In taekwondo-specific research, the most recent systematic review (2017) reported a predominance of lower-limb injuries and contusions, yet used heterogeneous denominators and largely reflected the pre-/early-PSS era [5]. Since then, numerous observational studies have investigated injury incidence, type, and severity in elite taekwondo athletes, often stratified by sex, setting, and level of competition. Despite this, inconsistency in injury definitions, exposure metrics, and methodology continues to hamper comparability and evidence synthesis [6,7].

Given these developments, a contemporary synthesis focused solely on high-performance taekwondo athletes post-PSS is essential to inform targeted injury-prevention strategies. The objective of this systematic review is therefore to summarize and critically appraise the current epidemiological evidence on injury incidence, anatomical location, type, causes, and severity in elite taekwondo athletes, with attention to distinctions across training versus competition and between male and female participants.

## Review

Methods

This systematic review was conducted in accordance with the Preferred Reporting Items for Systematic Reviews and Meta-Analyses (PRISMA) 2020 statement [8]. The protocol was developed a priori and followed a structured approach to ensure transparency and reproducibility, but no registration was performed. No registration in PROSPERO was performed.

Review Question

This systematic review aims to answer the following COCOPOP (Condition, Context, Population) question [9]: What is the current scientific evidence on the epidemiology of injuries - including their incidence, location, type, causes, and severity - among high-performance taekwondo athletes in competitive settings?

Eligibility Criteria

We included observational studies (cross-sectional, prospective, or retrospective cohort designs) published in English or Spanish between January 1, 2018, and December 31, 2024, that investigated injury epidemiology in high-performance taekwondo athletes. Studies were eligible if they reported at least one of the following outcomes: injury incidence, anatomical location, type, cause, or severity, and if participants were elite athletes (national or international competitive level).

We excluded studies that focused on a single specific injury without broader epidemiological context or reported mixed martial arts or other combat sports data without stratified taekwondo results. Studies were also excluded if they were inaccessible in full text after reasonable retrieval attempts or were systematic reviews, meta-analyses, theses, case reports, conference abstracts, or narrative reviews.

Information Sources and Search Strategy

Between February and March 2025, we systematically searched six databases: PubMed, Medline, Scopus, Web of Science, CINAHL, and SportDiscus. The search strategy combined Medical Subject Headings (MeSH) “Wounds and Injuries,” “Athletes,” and “Martial Arts,” with the free-text term “taekwondo,” linked with the Boolean operator AND. Full search strings for each database are presented in Table 1. A publication year filter (2018-2024) was applied to capture post-PSS era data. Reference lists of included studies and relevant reviews were also screened to identify additional eligible studies.

**Table 1:** Search equations

Base de datos	Ecuación de búsqueda
Pubmed	(("Martial Arts"[Mesh]) AND "Athletes"[Mesh]) AND "Wounds and Injuries"[Mesh] AND "taekwondo" [tw]
Medline	(MH "Athletes") AND (MH "Martial Arts") AND (MH "Wounds and Injuries") AND (TW "Taekwondo")
Scopus	(TITLE-ABS-KEY (athletes) AND TITLE-ABS-KEY (martial AND arts) AND TITLE-ABS-KEY (injuries) AND TITLE-ABS-KEY (taekwondo))
Web of Science	martial arts (Topic) AND athletes (Topic) AND injuries (Topic) AND taekwondo (Topic)
CINAHL	(MH "Martial Arts") AND (MH "Athletes") AND (MH "Wounds and Injuries") AND (TW "Taekwondo")
Sport Discus	(((DE "ATHLETES”) AND (DE "WOUNDS & injuries")) AND (DE "TAE kwon do")) AND (DE "MARTIAL arts")

Study Selection

All records were exported to a reference manager for duplicate removal. Two reviewers independently screened titles and abstracts against the eligibility criteria, followed by full-text review of potentially relevant articles. Disagreements were resolved through discussion or by consulting a third reviewer. Reasons for exclusion at the full-text stage were documented. The study selection process is illustrated in the PRISMA 2020 flow diagram.

Data Extraction

Two reviewers independently extracted data using a standardized form, including: study characteristics (country, sample size, sex, age), setting (training, competition, or both), observation period, injury definition, incidence measures, anatomical location, injury type, cause, severity, and denominators used for incidence rates (athlete-exposures, minutes, or hours). When available, data were stratified by sex and performance context.

Quality Assessment

The methodological quality and risk of bias of the included studies were assessed using the Joanna Briggs Institute (JBI) critical appraisal checklist for studies reporting prevalence and incidence data [10]. This tool evaluates internal validity and potential bias across eight domains, including sample selection, measurement reliability, and appropriateness of statistical analysis. Each domain was rated as “Yes,” “No,” “Unclear,” or “Not applicable.” Two reviewers performed the assessment independently; disagreements were resolved through consensus.

Data Synthesis

Due to the lack of standardisation in the definitions of outcomes and denominators a meta-analysis was not feasible. The heterogeneity in study designs, injury definitions, and incidence denominators was too high to justify quantitative pooling and obtain concise results. A narrative synthesis was conducted, organizing findings by outcome domain: incidence rates, anatomical location, injury type, cause, and severity. Results were also grouped by performance context (training vs. competition) and by sex when data were available.

Results

Study Selection

**Figure 1:**
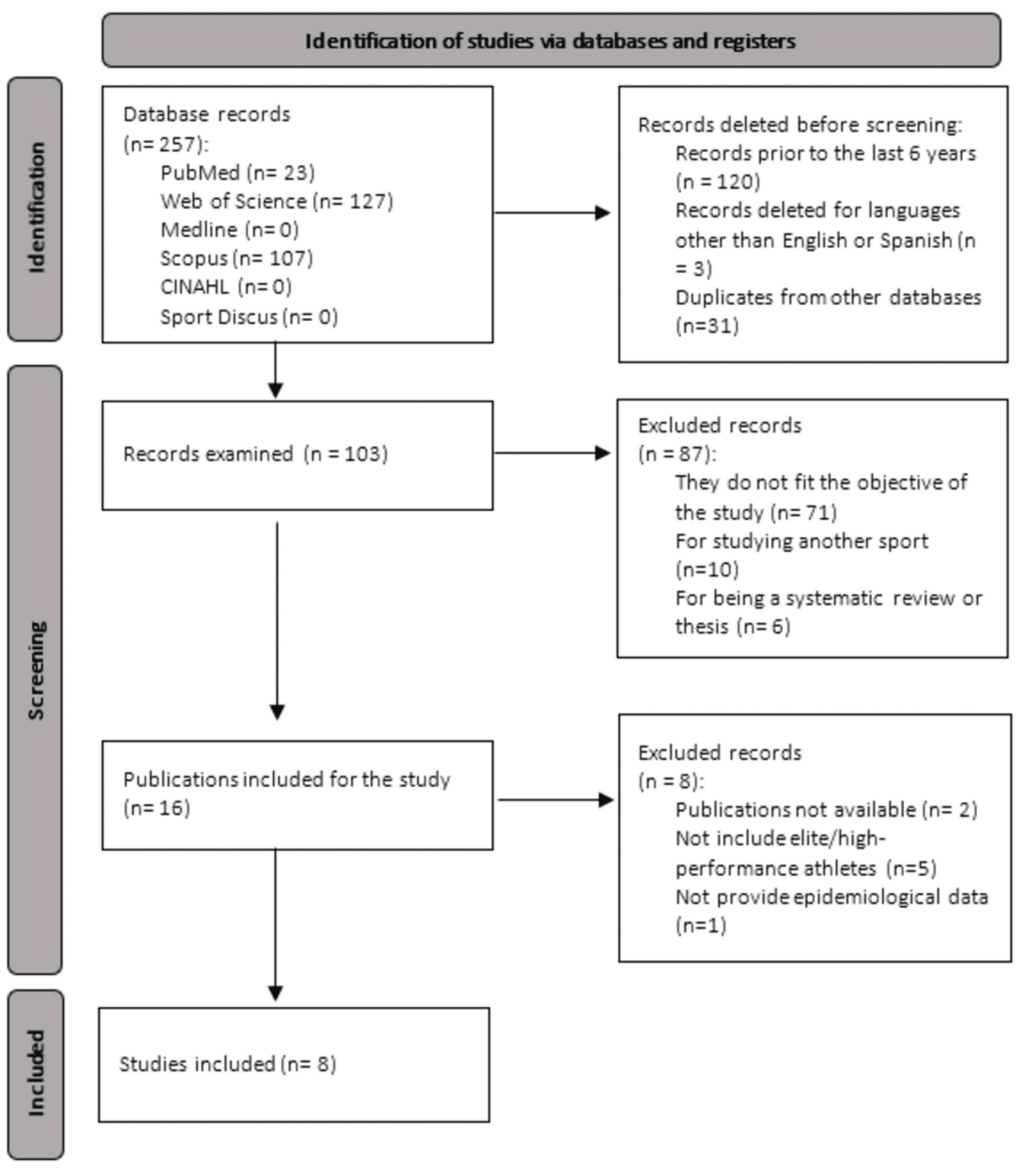
Preferred Reporting Items for Systematic Reviews and Meta-Analyses (PRISMA) 2020 Flow Diagram

The initial search retrieved 257 records. After duplicate removal, 103 records were screened by title and abstract, resulting in 16 full-text articles assessed for eligibility. Of these, two could not be retrieved in full text, five did not exclusively include elite/high-performance athletes and data could not be reliably extracted for this subgroup, and one was a qualitative study that did not provide epidemiological data. Eight studies met all inclusion criteria and were included in the qualitative synthesis (Figure 1).

Study Characteristics

The included studies, conducted between 2018 and 2024, encompassed 3,834 athletes competing at national, international, or Olympic levels. Study settings included national training centers, university training programs, and major international competitions such as the Senior and Junior World Championships and the Olympic Games. Sample sizes ranged from 66 to 971 athletes, with observation periods varying from five days to 10 years. Full details of each study are presented in Table 2.

**Table 2:** Characteristics of the included studies n: Sample size; SD: Standard deviation; M: men; W: women; y: year; d: days

Study	Country	Sample size (n)	Age (mean ± SD), range	Location	Observation
Geßlein et al., 2020 [11]	Germany	66; M: 37; W: 29	19.3 ± 4.2; M: 19.8 ± 4.5; W: 18.7 ± 3.7	National and international training and championships for athletes from the National Olympic Training Center	5 y
Han et al., 2023 [12]	South Korea	183: M: 129; W: 54	12-19 y	Training at universities in South Korea	1 y
Jeong et al., 2023 [13]	England	936; M: 544; W: 392	M: 22.5; W: 21.4	2019 Senior World Championship	5 d
Jeong et al., 2021a [14]	South Korea	971; M: 593; W: 378	M: 22.9 - 23.3; W: 22.3	2017 Senior World Championship	7 d
Jeong et al., 2021b [15]	Tunisia	889‚ M: 496; W:393	15-17 y	2018 Junior World Championships	7 d
Lystad et al., 2021 [16]	Australia	316	>15 y	Olympic Games 2008, 2012 and 2016	3 Olympic cycles
Park y Song, 2018 [17]	South Korea	283	< 18 y	South Korean National Training Center	10 y
Son et al., 2020 [18]	South Korea	285; M: 189; W: 96	17.10 ± 2.73; M: 17.15 ± 2.88; W: 17.00 ± 2.40	Training and championships in South Korea	1 y

Injury definitions varied, with most studies adopting time-loss criteria (≥1 day absence from training or competition) and a minority using medical attention definitions. Incidence rates were reported using different denominators, including athlete-exposures, athlete-minutes, or athlete-hours.

Methodological Quality

All included studies met most criteria of the JBI checklist for analytical cross-sectional studies. Seven studies clearly defined inclusion criteria, and all described subjects and settings in sufficient detail. Six studies identified potential confounding factors, and five implemented strategies to address them. All studies used valid and reliable outcome measures, and statistical analyses were deemed appropriate (Table 3).

**Table 3:** Methodological quality: Joanna Briggs Institute critical appraisal checklist. Methodological quality assessment for each study.

Authors	1	2	3	4	5	6	7	8
Geßlein et al., 2020 [11]	Yes	Yes	Unclear	Yes	No	No	Yes	Yes
Han et al., 2023 [12]	Yes	Yes	No	No	Yes	No	No	Unclear
Jeong et al., 2021a [14]	Yes	Yes	Yes	Yes	Yes	No	Yes	Yes
Jeong et al., 2021b [15]	Unclear	Yes	Yes	Yes	Yes	No	Yes	Yes
Jeong et al., 2023 [13]	Unclear	Yes	Yes	Yes	Yes	No	Yes	Yes
Lystad et al., 2021 [16]	Unclear	Yes	Yes	Yes	Yes	Yes	Yes	Yes
Park & Song, 2018 [17]	Yes	Yes	Yes	Yes	Yes	No	Yes	Yes
Son et al., 2020 [18]	Yes	Yes	Yes	Yes	Yes	Yes	Unclear	Yes

Injury Incidence

Injury incidence varied markedly among the included studies, reflecting differences in observation periods, competition levels, and incidence denominators. Across the eight studies, incidence ranged from 6.2 to 77.0 injuries per 1000 athlete-exposures and from 0.7 to 90.9 injuries per 1000 hours of exposure. The highest incidence was observed during international competitions, particularly the Senior World Championships, whereas the lowest rates were reported in multi-year surveillance at national training centers. The number of injuries and incidence rate can be seen in Table 4.

**Table 4:** Number of injuries and incidence rate. ♂: men; ♀: women; AEs: athlete exposures; MEs: minutes of exposure

Study	Number of injuries	Ratio/1000 hours	Injury incidence rate/1000 AEs	Injury incidence rate/1000 MEs
Geßlein et al., 2020 [11]	172	Competition: 94.98 Training: 15.00		
Han et al., 2023 [12]	1086: - ♂: 744 - ♀︎: 342		Teenagers: 4.43 - ♂: 3.93 - ♀︎: 5.34 University students: 3.13 - ♂: 2.91 - ♀︎: 4.44	
Jeong et al., 2023 [13]	60: - ♂: 41 - ♀︎: 19		33.08: - ♂: 38.46 - ♀︎: 25.40	7.63: - ♂: 9.64 - ♀︎: 5.26
Jeong et al., 2021a [14]	131: - ♂: 90 - ♀︎: 41		77.8 - ♂: 90.0 - ♀︎: 59.9	13.9 - ♂: 16.6 - ♀︎: 10.3
Jeong et al., 2021b [15]	67: - ♂: 40 - ♀︎: 27		38.55: - ♂: 4.15 - ♀︎: 32.25	6.93: - ♂: 7.40 - ♀︎: 6.34
Lystad et al., 2021 [16]	42		46.4	7.7
Park y Song, 2018 [17]	1466: - ♂: 577 - ♀︎: 889	Training: 5.9 - ♂: 4.6 - ♀︎: 7.1	26.1 - ♂: 20.5 - ♀︎: 31.7	
Son et al., 2020 [18]	336: - ♂: 210 - ♀︎: 126		Training: 4.79 - ♂: 4.18 - ♀︎: 6 Competition: 24.86 - ♂: 27.46 - ♀︎: 19.74	

Injury Location

**Figure 2:**
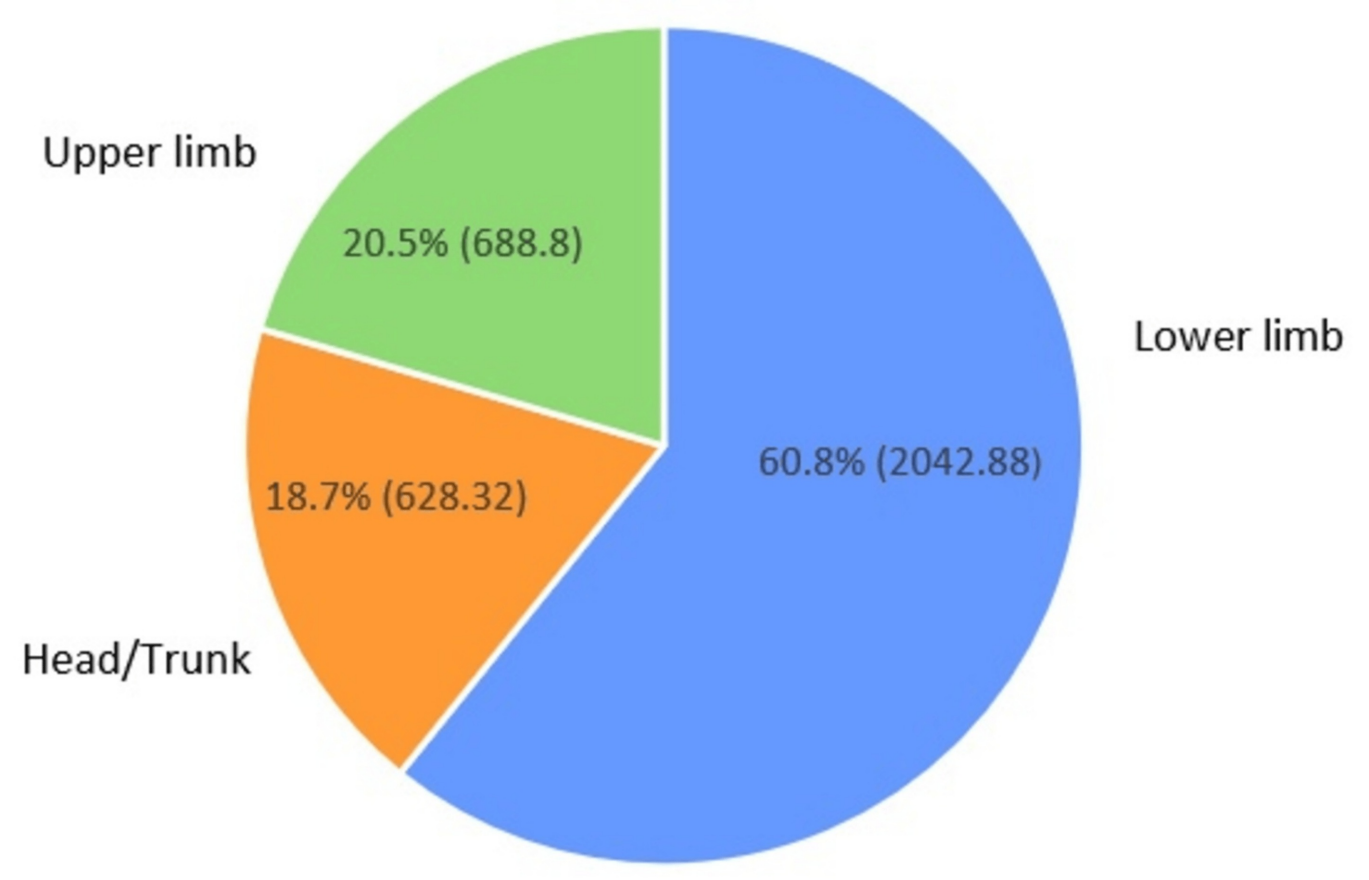
Anatomical distribution of injuries

Lower limb injuries were the most frequent across all eight studies, accounting for 51% (1713.6) to 80% (2688) of all reported cases. The knee, ankle, and thigh were consistently identified as the most affected regions. Upper limb injuries were less prevalent, representing 10% (336) to 25% (840) of cases, with the shoulder being the most frequently injured joint in this category. Head and trunk injuries were rare, generally accounting for less than 10% (336) of all injuries (Table 5, Figure 2).

**Table 5:** Injury Location %: percentage; n=: total number

Study	Number of injuries	Head and trunk %(n=)	Upper limbs %(n=)	Lower limbs %(n=)
Geßlein et al., 2020 [11]	172	10.46% (17.99)	33.14% (57) - Wrist and Hand: 29.65% (50.99)	56.4% (97) - Foot: 16.28% (15.79)
Han et al., 2023 [12]	1086	18.1% (196.56): - Head: 6.1% (11.99) - Face: 4.2% (8.25) - Waist, Lumbar: 6.6% (12.97)	27.5% (298.65): - Fingers: 15.9% (47.49) - Wrist: 5.7% (17.02)	54.5% (591.87): - Ankle: 15.2% (89.96) - Foot: 13.5% (79.909)
Jeong et al., 2023 [13]	60	38.33% (23): - Face: 25% (5.75)	18.33%: (11) - Hand: 11.67% (1.28)	43.33% (26): - Thigh: 10% (2.6) - Knee: 10% (2.6)
Jeong et al., 2021a [14]	131	32.06% (42): - Face: 24.42% (10.26)	23.7% (31): - Fingers: 6.1% (1.9) - Hand: 5.3% (1.65)	44.3% (58): - Knee: 16.8% (9.75)
Jeong et al., 2021b [15]	67	29.85% (20): - Face: 20.89% (4.18)	16.42% (11): - Fingers: 8.95% (0.98)	53.73% (36) - Feet: 16.42% (5.9)
Lystad et al., 2021 [16]	42	9.52% (4): - Head and Lumbar: 4.76% (0.19)	30.95% (13): - Wrist: 19% (2.47)	59.52% (25): - Thigh and Knee: 16.67% (4.17)
Park and Song, 2018 [17]	1466	20.4% (299): - Lumbar: 10.2% (30.5)	14.1% (207): - Shoulder and Clavicle 4.3% (8.89)	65.5% (960): - Ankle: 17.6% (169) - Knee: 13.2% (126.75)
Son et al., 2020 [18]	336	7.75% (26.04): - Lumbar: 5.06% (1.32)	17.87% (60.04): - Hand and Wrist: 14.29% (8.58)	74.11% (249.02): - Foot: 21.43% (53.36) - Ankle: 20.83% (51.87)

Figure 2 illustrates the anatomical distribution of injuries.

Injury Type and Mechanism

Sprains and strains were the most common injury types, together representing 35% (1176) to 60% (2016) of all reported injuries across the included studies. Contusions were also frequent, accounting for 15% (504) to 30% (1008) of cases. Overuse-related conditions, such as tendinopathies, were less prevalent but were more common in long-term surveillance studies than in event-based reports. Non-contact mechanisms predominated in most studies, often associated with repetitive loading or rapid changes in direction. Contact injuries, primarily resulting from collisions during competition, were less frequent but tended to result in more severe time loss (Table 6, Table 7, and Table 8).

**Table 6:** Injury type %: percentage; n=: Total number; ♂: men; ♀: women

Study	Contusions %(n=)	Sprain/ligaments tear %(n=)	Fractures %(n=)	Others
Geßlein et al., 2020 [11]	16.28% (28)	30.23% (52) (Includes other articular lesions)	21.51% (37)	-Muscle lesions: 7.56% (13) -Tendinous lesions: 6.39% (11)
Han et al., 2023 [12]	24.5% (266.07)	29.1% (316.02)	13.9% (150.95)	-Muscle strain/tear: 7.4% (80.36)
Jeong et al., 2023 [13]	55% (33): - ♂: 75.76% (25) - ♀︎: 24.24% (8)	11.67% (7): - ♂: 42.86% (3) - ♀︎: 57.14% (4)	10% (6): - ♂: 83.33% (5) - ♀︎: 16.67% (1)	-Concussion: 5% (3) - ♂: 100% (3) - ♀︎: 0% (0)
Jeong et al., 2021a [14]	33.6% (44.02) - ♂: 68.2% (30.02) - ♀︎: 31.8% (14)	16% (21) - ♂: 66.7% (14) - ♀︎: 33.3% (7)	23.7% (31.05) - ♂: 70.9% (22.01) - ♀︎: 29.1% (9.04)	-Tendinous lesions: 10.7% (14.02) - ♂: 64.3% (9.02) - ♀︎: 35.7% (5) -Concussion: 3.8% (4.98) - ♂: 80% (3.98) - ♀︎: 20% (1)
Jeong et al., 2021b [15]	55.22% (37) - ♂: 59.46% (22) - ♀︎: 40.54% (15)	10.45% (7) - ♂: 57.14% (4) - ♀︎: 42.86% (3)	7.46% (5) - ♂: 100% (5) - ♀︎: 0% (0)	Cutaneous lesions: 8.95% (6) - ♂: 33.33% (2) - ♀︎: 66.67% (4) Concussion: 4.47% (3) - ♂: 66.67% (2) - ♀︎: 33.33% (1)
Lystad et al., 2021 [16]	38% (16) (includes other muscle lesions)	33.3% (14)	14.28% (6)	-Neural lesions: 4.76% (2) -Tendinous lesions: 2.38% (1)
Park y Song, 2018 [17]	24.70% (362.1)	27.38% (401.4)	21.13% (309.76)	-Tendinous lesions: 7.74% (113.47)

**Table 7:** Injuries’ mechanism %: percentage; n=: Total number; ♂: men; ♀: women

Study	Contact %(n=)	Overuse %(n=)	Others %(n=)
Han et al., 2023 [12]	56% (608.1)	20.6% (223.72)	9.3% (100.1) no attention
Jeong et al., 2023 [13]	83.33% (50) - ♂: 74% (37) - ♀︎: 26% (13)	6.67% (4) - ♂: 25% (1) - ♀︎: 75% (3)	10% without contact (6) - ♂: 50% (3) - ♀︎: 50% (3)
Jeong et al., 2021a [14]	74% (96.94) - ♂: 73.19% (70.95) - ♀︎: 26.81% (25.99)	-	13.7% without contact (17.95) - ♂: 50% (8.97) - ♀︎: 50% (8.97)
Jeong et al., 2021b [15]	76.12% (51) - ♂: 58.82% (30) - ♀︎: 41.18% (21)	5.97% (4) - ♂: 75% (3) - ♀︎: 25% (1)	18 % (12) - ♂: 58.3% (7) - ♀︎: 41.6% (5)
Son et al., 2020 [18]	50.89% (171)	14.88% (50)	19.05% without contact (64)

**Table 8:** Time sports leave. %: percentage; n=: Total number; ♂: men; ♀: women; d: days

Study	No time off %(n=)	1 to 7 d %(n=)	7 to 28 d %(n=)	> 28 d %(n=)
Jeong et al., 2023 [13]	8.33% (5)	76.67% (46)	15% (9)	-
Jeong et al., 2021a [14]	34.4% (45)	35.1% (46)	30.5% (40)	-
Jeong et al., 2021b [15]	34.78% (23)	53.62% (36)	11.6% (8)	-
Lystad et al., 2021 [16]	45% (18.9)	22,5% (9.45)	10% (4.2)	22.5% (9.45)
Park y Song, 2018 [17]	-	79.4% (1164): - ♂: 38.9% (452.8) - ♀︎: 61.1% (711.2)	20.6% (302): - ♂: 41.1% (124) - ♀︎: 58.9% (178)	-
Son et al., 2020 [18] 336	24.4% (82)	28.6% (96)	20.83% (70)	26.2% (88)

Discussion

This systematic review synthesized evidence from eight observational studies assessing sports injuries in elite and high-performance athletes [11-18]. The findings confirm that injury incidence in this population varies substantially depending on study design, observation period, and calculation methods, with reported rates ranging from 6.2 to 77.0 injuries per 1,000 athlete-exposures [11,13,14] and from 0.7 to 90.9 injuries per 1,000 hours of exposure [12,17]. These values are generally higher than those observed in non-elite or recreational cohorts, likely reflecting the increased training load, competition intensity, and exposure frequency characteristic of high-performance athletes.

Lower limb injuries were consistently the most frequent across all studies, accounting for more than half of reported cases [11-18], coinciding with the results provided by other authors such as [2,19]. The ankle, knee, and thigh were the most commonly affected regions [12,15,17]. This is consistent with information provided by other authors, where the area most prone to injury was the ankle [20-22], followed by the knee [16,19].

Upper limb injuries, less prevalent overall, were predominantly localized to the shoulder [13,14], while head and trunk injuries, though relatively rare, remain clinically relevant, particularly due to their potential severity [13,16]. The predominance of sprains and strains, followed by contusions and overuse-related conditions [11,12,14,17], underscores the dual burden of acute and overuse injuries in elite taekwondo. Non-contact mechanisms were more frequently reported [12,13,17], highlighting the potential effectiveness of prevention programs focusing on neuromuscular control, movement efficiency, and load management.

These findings are broadly consistent with the most recent pre-PSS systematic review by Thomas et al. [5], which also identified a predominance of lower limb injuries, with contusions and sprains as the most common types. However, compared to that earlier synthesis, the present review suggests a relative decrease in head and facial injuries, likely attributable to the widespread implementation of electronic PSS during the past decade. This shift in injury distribution illustrates how regulatory and technological changes in competition can influence injury epidemiology, reinforcing the importance of continuous surveillance and updated evidence syntheses.

Sprains and strains were the most frequently reported injury types, followed by contusions and overuse-related conditions [11,12,14,17]. This pattern aligns with previous research on elite athletes and highlights the dual burden of acute injuries, often sustained during competition, and overuse injuries, more frequently observed in training environments [1]. In his study in 2016, Ji [22] stated that contusions were the most common type of injury during competition, which he justified by pointing to the characteristics of the sport itself, which involves very intense physical contact. Non-contact mechanisms predominated in most studies [12,13,17], suggesting that injury prevention programs targeting neuromuscular control, movement efficiency, and load management may be particularly beneficial in this population.

Methodological quality, as assessed by the JBI checklist, was generally moderate to high. Most studies clearly defined inclusion criteria and provided detailed descriptions of participants and settings [11,14-17]. All used valid and reliable measures for both exposure and outcome assessment. However, confounding factors were not consistently identified or controlled [12,18], and differences in injury definitions, surveillance periods, and data collection methods limit direct comparability across studies.

This review has several strengths. First, it exclusively included high-performance athletes, reducing heterogeneity related to skill level and training volume. Second, methodological quality assessment ensured a critical appraisal of the included evidence. Nonetheless, important limitations must be acknowledged. The relatively small number of studies and their concentration in specific sports limit the generalizability of findings to other high-performance disciplines [13,15]. The lack of uniform injury definitions and exposure metrics complicates pooled estimates and comparisons [12,16]. Furthermore, the absence of prospective, multicentre designs reduces the strength of causal inferences [11,17].

From a practical standpoint, these findings emphasize the need for targeted injury surveillance systems in elite sports, with standardized definitions, reliable exposure measures, and regular reporting. Prevention strategies should prioritize the most frequently affected anatomical regions and mechanisms, particularly lower limb non-contact injuries [12,15,17]. Future research should aim to expand the evidence base to a wider range of sports, incorporate longitudinal designs to track injury patterns over time, and evaluate the effectiveness of tailored prevention programs in elite athletic settings [13,16].

The main limitations of this review are the heterogeneity of injury definitions, the variable quality of reporting, and the relatively small number of high-quality studies focusing exclusively on elite athletes. The heterogeneity in study designs, injury definitions, and incidence denominators was too high to justify quantitative pooling and obtain concise results, and due to the lack of standardisation in the definitions of outcomes and denominators a meta-analysis was not viable.

## Conclusions

This systematic review highlights that elite and high-performance athletes are at a substantial risk of injury, with incidence rates exceeding those reported in non-elite populations. Lower limb injuries, particularly sprains and strains caused by non-contact mechanisms, predominate across sports, underscoring the need for targeted prevention strategies.

While methodological quality was generally moderate to high, variability in injury definitions, surveillance periods, and exposure measures limits comparability across studies. Future research should adopt standardized reporting methods, broaden the range of sports investigated, and evaluate prevention programs specifically tailored to the demands of elite competition.
